# Effect of educational intervention based on theory of planned behaviour on promoting preventive behaviours of oral cancer in rural women

**DOI:** 10.1186/s12905-023-02411-2

**Published:** 2023-05-09

**Authors:** Siamak Najafi, Fatemeh Mohammadkhah, Pooyan Afzali Harsini, Mojtaba Sohrabpour, Ali Khani Jeihooni

**Affiliations:** 1 Department of Internal Medicine, School of Medicine, Fasa University of Medical Sciences, Fasa, Iran; 2 Department of Community health, child nursing and aging, Ramsar School of Nursing, Babol University of Medical Sciences, Babol, Iran; 3 Department of Public Health, School of Health, Kermanshah University of Medical Sciences, Kermanshah, Iran; 4 Noncommunicable Diseases Research Center, Fasa University of Medical Sciences, Fasa, Iran; 5 Nutrition Research Center, Department of Public Health, School of Health, Shiraz University of Medical Sciences, Shiraz, Iran

**Keywords:** Education; women, Hookah use, Theory of Planned Behaviour (TPB)

## Abstract

**Background:**

Oral cancer has created an alarming situation around the world, and being the 16th most common cancer worldwide, it has become a global concern. The present study aimed to investigate the effect of educational intervention based on a theory of planned behavior (TPB) on promoting preventive behaviors of oral cancer in rural women.

**Methods:**

This quasi-experimental study was conducted on 120 female hookah users referring to rural health centers in Fasa and Shiraz city, Fars province, Iran. The subjects were divided into experimental and control groups by simple random sampling. The educational intervention for the experimental group consisted of 8 training sessions of 50 min. Both groups completed a questionnaire including demographic characteristics and constructs of TPB before and four months after the educational intervention. Data were analyzed using SPSS 22 software through independent t-test, chi-square, and paired t-test (p = 0.05).

**Results:**

The mean age of the experimental and control group subjects were 41.12 ± 8.86 and 40.63 ± 9.62 years, respectively (p = 0.185). The mean age of onset of hookah use in the experimental and control group was 24.16 ± 9.50 and 23.35 ± 9.44 years, respectively (p = 0.182). Also, before the educational intervention, there was a significant difference between the experimental and control groups in terms of knowledge (p < 0.189), attitude (p < 0.122), perceived behavioral control (p < 0.142), subjective norms (p < 0.236), behavioral intention (p < 0.126), oral cancer prevention behaviors (p < 0.108) and nicotine dependence (p < 0.218); however, four months after the educational intervention, there was a significant increase in the experimental group in all variables except nicotine dependence (p < 0.005).

**Conclusion:**

Due to the alarming situation of oral cancer and the prevalence of hookah use among women, educational programs based on TPB could effectively prevent hookah use and oral cancer.

## Background

Oral cancer has created an alarming situation around the world, and being the 16th most common cancer worldwide (with 377,700 cases in 2020), it has become a global concern. Oral cancer accounts for 48% of head and neck cancers [3]. Globally, the highest incidence of lip, tongue, and mouth cancer is in South-Central Asia and parts of Oceania (Papua New Guinea, Pakistan, and India). India has the highest rate of mouth and oral tongue cancer [4]. Approximately 25% of future cases of oral cancer are in these countries and are more common in men [5]; But both in men and women, the prevalence of oral cancer is decreasing [4]. Because oral cancer treatments have a dismal prognosis, prevention is essential. The 5-year survival rate for oral cancer is still less than 50% in the majority of nations [3] despite modern management techniques. On the other side, oral cancer therapies are quite costly. Consequently, it is crucial to start with preventative measures [1]. Effective oral cancer prevention strategies include reducing exposure to potential risk factors and getting screened for possibly malignant conditions (OPMD) [6]. Oral cancer is a complicated disease with several risk factors, including the use of tobacco and alcohol, chronic inflammation, ultraviolet (U.V.) radiation (for lip cancer), human papillomavirus (HPV) or Candida infections, immunosuppression, genetic susceptibility, and diet [7]. The two biggest risk factors for oral cancer are tobacco use, especially smokeless tobacco, and alcohol drinking [8]. Like cigarette tobacco, nicotine is also present in pipe and oral tobacco [9]. More than 4,000 compounds, sixty of which are carcinogenic, are found in tobacco smoke [5]. Hookah smoking is an ancient way of tobacco consumption that is growing around the globe and among many ethnicities. Many by-products in mainstream smoke (M.S.S.) are hazardous and carcinogenic. M.S.S. contains higher concentrations of some chemicals than cigarette smoke. Hookah smoking has adverse effects on the respiratory, cardiovascular, blood, reproductive, and fetal systems and on pregnancy. Additionally, hookah smoking has frequently been connected to the lung, oral, and nasopharyngeal cancers. By subjecting the user to carcinogens in hookah smoke, continuous mechanical stimulation, and infections brought on by sharing the hookah’s mouthpiece, hookah use increases the risk of oral cancer [10]. The head, body, water bowl, hose, and mouthpiece are the five main elements of a modern hookah. Charcoal that is burning is placed on the head. Smoke goes to the user through the hose when they inhale, sucking it from the head through the body and into the water bowl [10]. Currently, one of the most avoidable causes of death in the world is tobacco use. It is predicted that tobacco usage will cause the deaths of 5 million people annually by 2030 and 8 million by then. Notably, this mortality occurs in low- and middle-income countries [11]. Middle Eastern youth frequently use hookah, and its use is quickly spreading across the globe. The reports on hookah use around the world are as follows: In Australia (11,4% of adolescents), Canada (10% of students), the United States (8.4% of 152 college and university students), and Arab nations (9–15% of 13–15-year-old students) [12]. High rates of hookah use [32.3%] are also observed among Iranian schoolchildren and college students [13]. In research by Bashirian et al. (2021), approximately 32.2% of participants had a single hookah smoking experience during their lifetime, and 20.4% were current consumers. The majority of individuals smoked hookah at their friends’ residences (45.8%) and with their friends (47.2%) [14].

Notable is the global surge in hookah usage among women and girls. Hookah is used by 7.8% of Jordanian women and 50% of Iranian women smokers. In Lebanon, the United Arab Emirates, and Iran, females aged 13 to 15 use hookahs often.

13.98% of women reported using hookahs on average. Women’s hookah smoking is influenced by awareness, positive attitudes, family and friends, sociopolitical variables [15], social norms, harm perceptions [16], a smoker friend, a low-risk perception, sociocultural acceptability of hookah, simple access, and the absence of prohibitive regulations [17]. A study by Dadipour et al. (2020) found that hookah smoking has been more common in Iran over the past few decades, particularly in the country’s south [18]. Many aspects of hookah smoking have not been thoroughly studied, despite the 400-year history of the practice and its current resurgence in popular culture. The development of hookah prevention methods is one aspect of hookah use that needs more research, according to the World Health Organization [19]. Currently, it is thought to be best practice to use particular theories when creating and evaluating treatments for behavior change (Connor & Norman, 2015). Choosing and defining constructs is one advantage of putting theory-based treatments into practice. Additionally, gathering empirical evidence from theory-based therapies might help understand underlying problems. The theory’s described processes suggest that therapies work. For creating interventions, a variety of behavior modification theories are available. TPB is a well-known model that has drawn much interest in health behavior research. It explains health-related behaviors like physical activity, diet, alcohol usage, and smoking [20]. The intention to engage in action is predicted by attitude, subjective norm, and perceived behavioral control, following TPB theory [21]. There have not been many studies on hookah use in Iran. Most studies in Iran only show the prevalence of hookah use. There is a lack of studies on the factors affecting hookah use and the impact of a theory-based educational program [22]. In a study by Sabzmakan et al., a positive attitude and misconceptions and regarding hookah usage were the primary determinants of the intention not to quit [26]. In addition, in other studies verified the correlation between misunderstandings regarding hookah use and hookah smoking behavior [33–34].Based on research findings, educational programs designed Based on Theory of Planned Behaviour to prevent and regulate tobacco, cigarettes, and hookah are beneficial [22, 23, 29–31].

On the other hand, due to the rapid increase in hookah use among Iranian women and the harmful effects of smoking on women’s health, including reproductive health, intervention programs are necessary to reduce hookah use among this group. Given the WHO’s emphasis on further research into the development of hookah prevention strategies [10], the role of factors such as knowledge, attitudes, and subjective norms in hookah use among women [15, 16], the role of TPB in explaining different health behaviors, the effect of interventions based on this theory on reducing hookah smoking behavior [23], and the relationship between hookah smoking and oral cancer [11], the present study aimed to investigate the effect of educational intervention based on a theory of planned behavior (TPB) on promoting preventive behaviors of oral cancer in rural women.

### Question of the study

Is the intervention based on a theory of planned behavior (TPB) effectively promoting preventive behaviors of oral cancer in rural women?

## Methods

This quasi-experimental study was conducted on 120 female hookah users referring to rural health centers in Fasa and Shiraz city, Fars province, Iran 2021–2022. Two centers were randomly selected out of rural health centres, 2 centres were randomly selected (one as the control group and the other as the intervention group), and a healthy house was selected from each center.

First, from among all rural health centers in Fasa, Iran (6 centers), 2 centers were randomly selected. Then, participants were recruited using a sampling method from each center(60 mothers in each group) and then were randomly divided into intervention (n = 60) and control (n = 60) groups according to their household number registered in their health file, according to the inclusion criteria [Inclusion criteria: 20 years old and older women with no oral cancer. Exclusion criteria: the case study’s unwillingness to participate in the research, failure to answer questions.], the female hookah users were invited to participate in the study and attend the health center.

### Sampling

The subjects were selected using a simple sampling method. According to the studies by Hassani et al. [22] and Jovini et al. [24], the sample size with 95% confidence, 80% test power, and standardized difference of 0.5 was calculated as 120.

### Inclusion and exclusion criteria

Inclusion criteria were women ≥ 20 years of age who smoked hookah once a day for at least one year and obtained written consent to participate in the study.

**Fig. 1:**
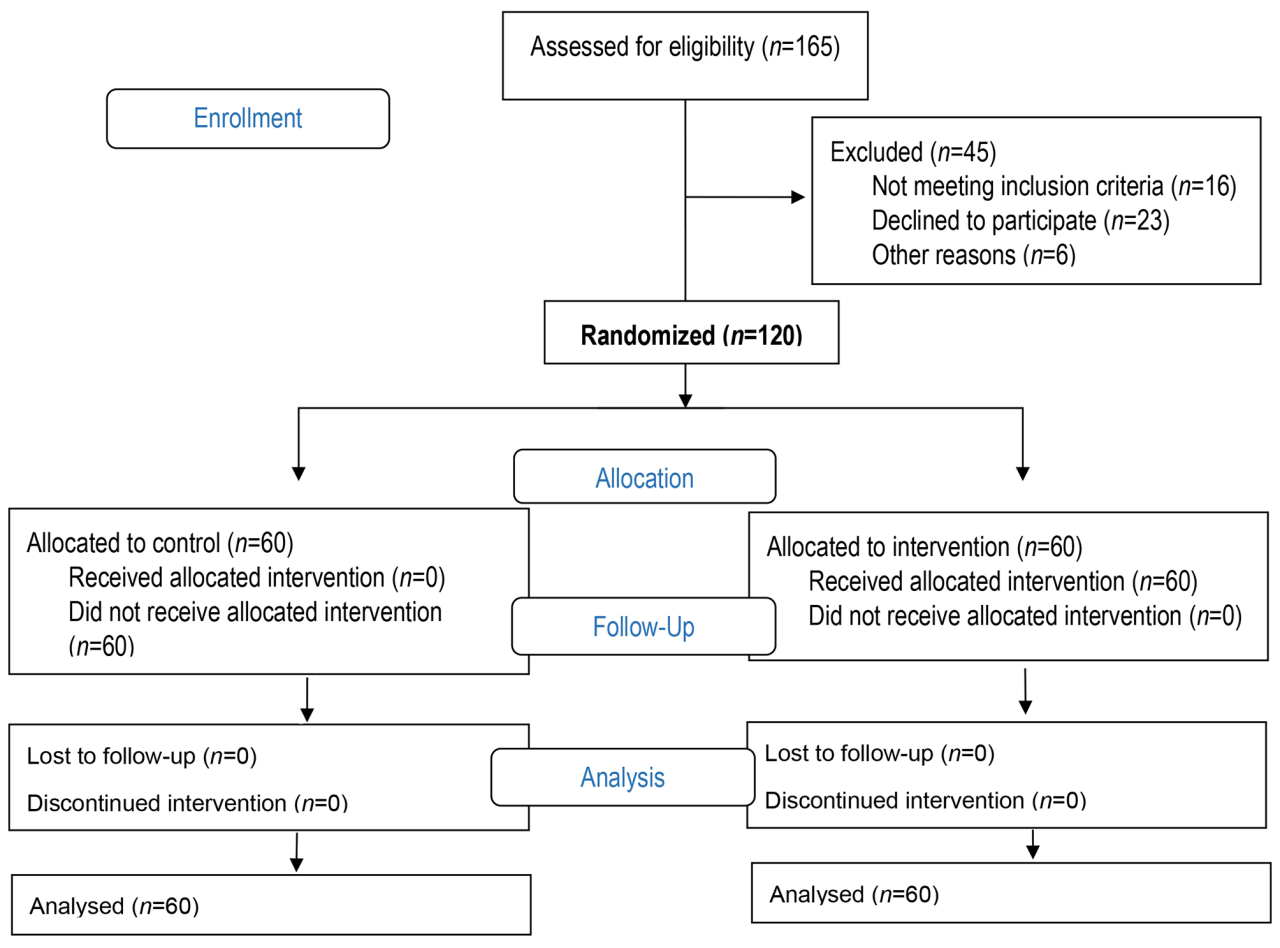
Flow chart of study

Exclusion criteria included mental or physical illness, absence from more than one session, and unavailability due to a change of residence. Figure 1 presents the study flow chart.

### Data collection tool

Data collection tools in this study included demographic characteristics (marital status, education, household income, history of smoking, family history of oral cancer, history of alcohol use, family history of hookah use, type of hookah used) and TPB questionnaire (knowledge, attitude, subjective norms, perceived behavioral control, behavioral intention).

Data were collected through a questionnaire through interviews whose validity and reliability was confirmed in other studies [22–27].

### TPB questionnaire

This questionnaire measured knowledge, attitude, perceived behavioral control, subjective norms, behavioural intention, and oral cancer prevention behaviors with 15, 12, 10, 8, and 12 questions based on a 5-point Likert scale, respectively. Nicotine dependence was also measured by 19 questions of the Nicotine Dependence Schiffman Scale (NDSS) based on a 5-point Likert scale [28].

The experimental and control groups completed the questionnaire before and four months after the educational intervention.

### Educational intervention

Eight 50-minute training sessions using lectures, group discussions, Q&A, instructive films and photos, PowerPoint, and a WhatsApp group comprised the educational intervention for the experimental group. The training program for health promotion and education was run by a Ph.D. with the assistance of an oral pathologist, an oncologist, and a psychologist.

These sessions covered topics such as the epidemiology of smoking, various tobacco product types, hookah use among different age groups, the history of hookah use and smoking-related deaths, the impact of hookah on oral cancer, symptoms, complications, and disease diagnosis, the prevalence of oral cancer, and risk factors like hookah use, smoking, alcohol use, and sun exposure. Additionally, behavioral ideas concerning hookah use and the advantages of quitting were taught through conversation and brainstorming. Through group discussion, controlled beliefs and factors that support quitting or reducing hookah use, as well as behaviors to prevent oral cancer, were taught; these included adjusting perceived power to reduce hookah use, declining invitations from others to smoke hookah, avoiding smoking during fun and happiness, and avoiding smoking when tired or angry. Strategies to enhance the perceived behaviors of individuals to stop using hookah included asking them to gradually cut back on their use, encouraging them to do so, and offering a successful example of cutting back and quitting. The ugliness of hookah smoking has significantly decreased because it is most frequently used in social settings with family and friends. Family members, healthcare professionals, doctors, and dentists also spoke about the advantages of quitting hookah and fostering more optimistic attitudes. It was stressed that family and friends might serve as useful, subjective norms for hookah use. It was decided to use an oral cancer patient as a model for presenting risk factors, symptoms, and side effects. A balanced diet, refraining from using tobacco products, limiting or eliminating alcohol use, using sunscreen on the face and lips, especially when exposed to direct sunlight, and other oral cancer prevention practices and their advantages were also covered. Subjects were divided into groups of 20 (6 groups of 20), and the role of friends and peers in accepting oral cancer prevention behaviors, especially quitting hookah use, was emphasized. In the end, the previous sessions were reviewed, and an educational booklet was delivered to the subjects. A WhatsApp group was formed to exchange information, and three educational and motivational messages were sent to people per week. Two follow-up sessions were held one month and two months after the educational intervention.

### Ethical considerations

The present study was approved by the Ethics Committee of Shiraz University of Medical Sciences [I.R.SUMS.SCHEANUT.REC.1400.110]. Written consent was obtained from the participants, and they were assured that their information would be treated strictly confidential.

Both experimental and control groups participated from the beginning to the end of the study, and no one was excluded. The control group did not receive any training program and was only invited to a special session to complete the questionnaire. At the end of the study, to comply with ethical standards, a training session was held for the control group on oral cancer and the importance of quitting hookah.

### Data analysis

Data were analyzed using SPSS 22 software through independent t-test, chi-square, and paired t-test (p = 0.05).

## Results

Under the auspices of Fasa rural health facilities, the present study was done on 120 female hookah users (60 in the experimental group and 60 in the control group). The average ages of the experimental and control groups were 41.12 ± 8.86 and 40.63 ± 9.62 years, respectively (p = 0.185). The average age of hookah users in the experimental and control groups was 9.91 ± 6.50 versus 23.9 ± 35.44 years, respectively (p = 0.182). The average hookah use history of the experimental and control groups was 15.39 ± 8.68 and 14.7 ± 69.14 years, respectively (p = 0.191). The independent t-test revealed a statistically significant difference between the experimental and control groups in terms of the average household size, which was 4.66 ± 1.12 versus 4.52 ± 1.44 (p = 0.159). Other demographic factors between the two groups were not significantly different (Table 1).

**Table 1:** Demographic characteristics of the participants:

Variables		Experimental group		Control group		p-value
		Number	Percentage	Number	Percentage	
Household income	≤ 30 million Rials	46	33.38	38	67.31	0.118
	30–60 million Rials	58	33.48	62	67.51	
	≥ 60 million Rials	16	34.13	20	66.16	
Education	Illiterate	4	3.34	2	1.67	0.124
	Primary school	22	18.33	28	23.33	
	Secondary school	46	38.33	38	31.67	
	High school	40	33.33	40	33.33	
	College	8	6.67	12	10	
Marital status	Single	8	6.67	10	8.33	0.112
	Married	101	84.16	106	88.33	
	Divorced	5	4.17	1	0.84	
	Widowed	6	5	3	2.50	
Family history of oral cancer	Yes	4	3.33	6	5	0.202
	No	116	96.67	114	95	
History of smoking	Yes	72	60	64	53.33	0.139
	No	48	40	56	46.67	
History of alcoholism	Yes	22	18.33	18	15	0.192
	No	98	81.67	102	85	
Family history of hookah use	Yes	114	95	110	91.67	0.107
	No	6	5	10	8.33	
Type of hookah used	Fruit	64	53.33	60	50	0.208
	Traditional	56	46.67	60	50	

At the beginning of the study, there was no significant difference between the experimental and control groups regarding knowledge, attitude, perception of behavioral control, subjective norms, behavioral intentions, behaviors related to oral cancer prevention, or nicotine dependence. Four months following the educational intervention, there was a substantial increase in the experimental group in each situation except nicotine dependency (Table 2).

**Table 2:** Mean score of TPB constructs, oral cancer prevention behaviors, and Nicotine dependence of the participants:

Variables	Group	Before the intervention	4 months after the intervention	mean difference	p-value
Knowledge	Experimental	7.44 ± 1.52	13.14 ± 1.22	5.7 ± 0.3	0.001
	Control	6.98 ± 1.64	7.34 ± 1.37	0.36 ± 0.27	0.195
	p-value	0.189	0.001	-	
Attitude	Experimental	28.76 ± 4.67	52.48 ± 4.18	23.72 ± 0.49	0.001
	Control	30.45 ± 4.62	32.76 ± 4.58	2.31 ± 0.04	0.104
	p-value	0.122	0.001	-	
Perceived behavioral control	Experimental	25.55 ± 3.81	41.97 ± 3.95	16.42 ± 0.14	0.001
	Control	24.70 ± 3.69	26.80 ± 3.73	2.1 ± 0.04	0.101
	p-value	0.142	0.001	-	
Subjective norms	Experimental	13.52 ± 2.48	26.58 ± 2.39	13.06 ± 2.09	0.001
	Control	13.72 ± 2.56	13.78 ± 2.68	0.06 ± 0.12	0.248
	p-value	0.236	0.001	-	
Behavioral intention	Experimental	6.33 ± 0.96	10.71 ± 0.94	4.38 ± 0.02	0.001
	Control	6.57 ± 0.71	6.66 ± 0.73	0.09 ± 0.02	0.172
	p-value	0.126	0.001	-	
Oral cancer prevention behaviors	Experimental	5.28 ± 0.57	10.94 ± 0.72	5.66 ± 0.15	0.001
	Control	5.56 ± 0.62	5.58 ± 0.67	0.02 ± 0.05	0.232
	p-value	0.108	0.001	-	
Nicotine dependence	Experimental	82.95 ± 6.89	30.16 ± 6.28	52.79 ± 0.61	0.001
	Control	85.76 ± 6.38	82.39 ± 6.70	3.4 ± 0.32	0.202
	p-value	0.218	0.001	-	

## Discussion

As Sadeghi et al. (2019), Little et al. (2016), and Xavier et al. (2019), the experimental group showed significant increases in knowledge, attitude, perceived behavioral control, subjective norms, behavior intentions, and oral cancer prevention activities (2017) [29–31].

In Sadeghi et al. (2019) study with the aim of investigating review of studies aimed at preventing consumption Hookah was done. The research results show the usefulness of theory-based interventions such as TPB theory to prevent hookah consumption [29].

Also in Little et al. (2016) study with the aim of investigating Efficacy of a brief tobacco intervention for tobacco and nicotine containing product use in the U.S. Air Force, educational programs designed to regulate tobacco and nicotine containing product use was beneficial [30].

In Xavier et al. (2019)study with the aim of investigating education against tobacco, for secondary schools in Brazil was done. The research results show the usefulness of interventions to prevent smoking consumption [31].

Based on these research findings, educational programs designed to prevent and regulate tobacco, cigarettes, and hookah are beneficial. Four months after the educational intervention, the experimental group’s mean score on knowledge regarding hookah use prevention behaviors increased dramatically. Using PowerPoint and WhatsApp boosted the experimental group’s understanding. In a study by Khani Jeihooni et al. (2020) with the aim of investigating Effect of Educational Intervention based on TPB on Preventing Water Pipe Smoking in Secondary School Students, comparable results were obtained, and TPB-based intervention dramatically boosted target group knowledge [23]. In a study by Hassani et al. (2019), with the aim of investigating Effect of Educational Intervention Based on Theory of Planned Behaviour on the Reduction of Water Pipe Smoking in Women, the intervention group increased significantly in all constructs [22]. The experimental group’s mean attitude score increased significantly Four months following the educational intervention. In these training sessions, attitudes and behavioral beliefs regarding hookah were taught through brainstorming, and the good effects of quitting were discussed through conversation, resulting in a favorable attitude toward oral cancer prevention behaviors. In a study by Dehdari et al. (2013), with the aim of investigating Effect of education intervention using theory of planned behaviour on Hookah smoking in the male college students, the intervention group’s mean attitude score increased significantly, which is consistent with our findings [32]. The experimental group’s mean attitude score increased significantly Four months following the educational intervention.

The association between attitude and hookah smoking behavior is clear [23], as in a qualitative study by Sabzmakan et al. (2020), a positive attitude and misconceptions regarding hookah usage were the primary determinants of the intention not to quit [26]. In addition, studies by Abedini et al. (2014) and Elias et al. (2017) verified the correlation between misunderstandings regarding hookah use and hookah smoking behavior [33–34].

The experimental group’s mean score of perceived behavioral control considerably increased Four months after the educational intervention. In these training sessions, various strategies were used, including encouraging participants to gradually reduce their hookah use as a successful model for reducing and quitting smoking to improve their perception of behavioral control. As hookah use was gradually reduced, the experimental group’s mean score for perceived behavioral control increased. There was a statistically significant change in perceived behavioral control between the experimental and control groups after the intervention in a study of a similar nature by Shahriyarimoghadam et al. (2020) [35]. In this study with the aim of investigating Effect of Community-Based Health Education Campaign Based on TPB on Reduction of Hookah Smoking Among Women, There was a statistically significant change in perceived behavioral control between the experimental and control groups after the intervention.

The experimental group’s mean score of subjective norm considerably increased Four months after the educational intervention that is Consistent with Ezzati et al. (2015) [36] with the aim of investigating Effect of educational intervention based on theory of planned behaviour and reduced water pipe smokingamong women above 15 (yrs.), the mean subjective norm score grew significantly in the experimental group following the educational intervention. This study investigated the advantages of quitting hookah and the role of friends and family as subjective norms in the presence of a family member, a health professional, a physician, and a dentist. Inviting a person with oral cancer to talk as a model about oral cancer and its risk factors, symptoms, and complications raised the mean score of subjective norms in the intervention group. Numerous research has explored the effect of subjective norms on tobacco use behavior [35, 37–39], including Sabzmakan et al. (2020), Jawad et al. (2015), and Noonan et al. (2012), where peers and family members were highlighted as the primary determinants of not quitting [26, 40, 41]. According to these results and the result of the present study, it is possible to improve the subjective norms in the consumption of hookah we should have an important role in preventing and controlling people’s hookah smoking behavior.

Four months after the educational intervention, there was a significant increase in the experimental group’s mean score of behavioral intention, which is consistent with the results of studies by Khani Jeihooni and Athamneh et al. (2020) [23, 42] which shows the effect of educational intervention on improving behavioral intention in the intervention group.Concerning the relationship between behavioral intention and tobacco use [37, 39, 43–46], Therefore, it is possible to prevent and control the use of hookah by influencing the behavioral intention of hookah users. our findings are consistent with those of Momenabadi (2018) [47], Lareyre et al. (2021) [20], and Khani Jeihooni et al. (2020) [23].

The TPB is one of the effective behavior modification models [48]. Consistent with the findings of Tahmasebi et al. (2017) [49], the results of our investigation demonstrated a significant reduction in nicotine dependence among the experimental group. In the present study, the subjects were instructed on oral cancer prevention behaviors and their benefits, such as avoiding tobacco products, reducing or avoiding alcohol consumption, consuming a balanced diet, and using sunscreen on the face and lips, especially when exposed to direct sunlight, which resulted in a significant increase in the mean score of behavioral control in the experimental group and reduction in nicotine dependence among the experimental group. One limitation of this study is that unwillingness to talk about a problem(hookah use) could have prevented some women from attending the study. On the other hand, the population in this study is considered a group of rural women [with particular geographical, cultural, and ethnic conditions]. Consequently, the findings of this study can be generalized to other geographical circumstances.

## Conclusion

The 16th most prevalent cancer in the world is lip and mouth cancer. On the other side, smoking hookah raises your risk of developing mouth cancer. Iranian ladies are hardly an exception to the notable rise in hookah use among women worldwide. The W.H.O. states that the creation of hookah preventive measures is one area of hookah use that requires additional study. Using theory while creating and assessing behavior change treatments is a good idea. The Theory of Planned Behavior is a well-known concept that has drawn significant interest in studying health behavior (TPB). The current study, which involved 120 female hookah smokers, was carried out by rural health clinics. According to the findings, the experimental group significantly outperformed the control group regarding knowledge, attitude, perceived behavioral control, subjective norms, behavioral intentions, oral cancer prevention behaviors, and nicotine dependence Four months after the educational intervention. Based on the findings of this study, educational programs avoiding hookah use and oral cancer based on the TPB could be very successful due to the grave condition of oral cancer and the prevalence of hookah use among women. Also, These interventions may be expanded to other cities and rural areas by involving government agencies. Policymakers should provide health laws related to oral cancer prevention behaviors and the necessary financial support to encourage the adoption of oral cancer prevention behaviors.

## Data Availability

The datasets used and/or analyzed during the current study can be made available by the corresponding author on reasonable request.

## Abbreviations

TPB: Theory of Planned Behavior
